# Protocol for an effectiveness- implementation hybrid trial to assess the effectiveness and cost-effectiveness of an m-health intervention to decrease the consumption of discretionary foods packed in school lunchboxes: the ‘SWAP IT’ trial

**DOI:** 10.1186/s12889-019-7725-x

**Published:** 2019-11-12

**Authors:** Rachel Sutherland, Alison Brown, Nicole Nathan, Lisa Janssen, Renee Reynolds, Alison Walton, Nayerra Hudson, Amelia Chooi, Serene Yoong, John Wiggers, Andrew Bailey, Nicole Evans, Karen Gillham, Christopher Oldmeadow, Andrew Searles, Penny Reeves, Chris Rissel, Marc Davies, Kathryn Reilly, Brad Cohen, Tim McCallum, Luke Wolfenden

**Affiliations:** 1 Hunter New England Population Health, Locked Bag 10, Wallsend, NSW 2287 Australia; 2 School of Medicine and Public Health, University of Newcastle, University Drive, Callaghan, NSW 2308 Australia; 3 Hunter Medical Research Institute, 1/Kookaburra Circuit, New Lambton Heights, NSW 2305 Australia; 4 Priority Research Centre for Heath Behaviour, University of Newcastle, University Drive, Callaghan, NSW 2308 Australia; 5 Mid North Coast Local Health District, P.O. Box 126, Port Macquarie, NSW Australia; 6 Central Coast Local Health District, 4-6 Watt Street, Gosford, NSW 2250 Australia; 7 New South Wales Ministry of Health, NSW Office of Preventive Health, Liverpool, NSW Australia; 8 Skoolbag, Hamilton, NSW Australia; 9 New South Wales Department of Education, Sydney, NSW Australia

**Keywords:** Childhood obesity, Lunchboxes, Children, Child nutrition, M-health, Schools, Hybrid, Randomised controlled trial, Technology

## Abstract

**Background:**

At a population level, small reductions in energy intake have the potential to contribute to a reduction in the prevalence of childhood obesity. In many school systems, there is the potential to achieve a reduction in energy intake through modest improvements in foods packed in children’s school lunchboxes. This study will assess the effectiveness and cost-effectiveness of a multi-component intervention that uses an existing school-based communication application to reduce the kilojoule content from discretionary foods and drinks consumed by children from school lunchboxes whilst at school.

**Methods:**

A Type I hybrid effectiveness-implementation cluster randomised controlled trial will be conducted in up to 36 primary schools in the Hunter New England, Central Coast and Mid North Coast regions of New South Wales, Australia. Designed using the Behaviour Change Wheel, schools will be randomly allocated to receive either a 5-month (1.5 school terms) multi-component intervention that includes: 1) school lunchbox nutrition guidelines; 2) curriculum lessons; 3) information pushed to parents via an existing school-based communication application and 4) additional parent resources to address common barriers to packing healthy lunchboxes or a control arm (standard school practices). The study will assess both child level dietary outcomes and school-level implementation outcomes. The primary trial outcome, mean energy (kJ) content of discretionary lunchbox foods *packed in children’s lunchboxes*, will be assessed at baseline and immediately post intervention (5 months or 1.5 school terms). Analyses will be performed using intention to treat principles, assessing differences between groups via hierarchical linear regression models.

**Discussion:**

This study will be the first fully powered randomised controlled trial internationally to examine the impact of an m-health intervention to reduce the mean energy from discretionary food and drinks packed in the school lunchbox. The intervention has been designed with scalability in mind and will address an important evidence gap which, if shown to be effective, has the potential to be applied at a population level.

**Trial registration:**

Australian Clinical Trials Registry ACTRN:12618001731280 registered on 17/10/2018. Protocol Version 1.

## Background

Excessive weight gain is a leading contributor to the disease burden in Australia and internationally [1, 2]. As such, addressing overweight and obesity and the promotion of healthy eating habits in children and adults is a public health priority [3]. There is evidence that high intake of energy-dense, nutrient poor foods, referred to as ‘discretionary foods’, is a risk factor for obesity [4, 5]. As consumption patterns of these foods in childhood predicts intake in adulthood, establishing healthy dietary patterns in children may help to reduce the future burden of nutrition related chronic disease [6].

Accordingly, leading health authorities and governments internationally support schools to implement healthy nutrition policies and practices to limit the provision and consumption of discretionary foods among school-aged children [7, 8]. Whilst there have been considerable efforts internationally to improve the availability and purchase of healthy foods provided or sold at school via cafeterias [9] and canteens [10], most of the food consumed (86%) at school by students in many countries is brought from home via a lunchbox [11–13]. However, often the foods packed within lunchboxes are high in energy [14]. School lunchboxes in Australia, for example, contain an average of approximately 3000 kilojoules (kJ) and more than 3 serves (1200 kJ) of discretionary foods [14, 15]. Similarly, lunchboxes in the UK and USA also contain excessive discretionary foods [16, 17] with a study conducted in the UK reporting that the majority of lunchboxes (82%) had discretionary choices such as savoury snacks and confectionary [16]. A study in the USA with 129 Year 5 and 6 students reported children with packed lunches consumed significantly more sugar, saturated fat, sodium and overall energy than those consuming school provided meals [17]. At a population level, small reductions in energy intake, equivalent to approximately 420 kJ across a whole day, have the potential to reduce the prevalence of childhood obesity [18]. Given the excessive energy content and frequent inclusion of discretionary food items in children lunchboxes, modest reductions in the packing of these items in lunchboxes is likely to make an important contribution to achieving such a reduction in energy intake [18, 19].

Whilst evidence and Government policy support improving the nutritional quality of foods packed for children, there is currently little evidence regarding interventions targeting improvement in the nutritional quality of school lunchboxes. A systematic review, conducted by the research team, of interventions targeting the nutritional quality of school and centre-based care lunchboxes identified only ten trials including eight randomised controlled trials and two with quasi-experimental designs, [20] all of which tested approaches to supporting parents to pack healthy foods. All studies included a parental knowledge component via pamphlets, posters, workshops and newsletters, with the majority also including a child knowledge strategy via videos, games and curriculum. Interventions also included physical resources such as lunch packs and containers (*n* = 4) and incentives for children to try fruits and vegetables (*n* = 2) [20]. The interventions reported considerable challenges in accessing and engaging parents through these means. The review found inconsistent impacts between lunchbox interventions in relation to the packing of discretionary foods and beverages [20]. A meta-analysis on four of the included studies highlighted a moderate increase in the provision of vegetables, but no change to fruit. However, there were limitations of included studies in the review as consumption across the entire day was not recorded for the majority of studies which make it difficult to determine any displacement of food serves outside of school hours. As such, previous studies have typically reported little impact of lunchbox interventions on child dietary intake [20].

Evidence is emerging regarding the effectiveness of mobile text messaging [21] and app based interventions [22] as a highly scalable, effective and cost-effective intervention approach for improving a variety of health behaviours. To examine proof of concept and feasibility of such an intervention in improving the packing of healthy lunchboxes, a pilot randomised controlled trial (RCT) was undertaken in 12 primary schools [23]. The ‘SWAP IT’ pilot intervention used an existing school mobile communication app, already adopted by schools to communicate with parents, to provide information via push-notifications and messages to encourage parents to ‘swap’ discretionary foods from their child’s lunchboxes to healthier alternatives consistent with the Australian Dietary Guidelines (‘everyday’ foods). The pilot found 68% of 2100 parents opened the messages via the school mobile communication app, and 84% reported the messages provided were useful [23]. Observational measurement of children’s lunchboxes (*n* = 1462) following the pilot intervention found an increase in the mean energy from everyday foods packed in the lunchbox (83.13 kJ, CI = 2.65, 163.61, *p* = 0.04), and a reduction in both the mean energy from discretionary foods (− 211.61 kJ, CI = -426.16, 2.95, *p* = 0.05) and mean total energy of foods packed in lunchboxes (− 131.61 kJ, CI = -317.26, 54.05, *p* = 0.16) among students attending intervention schools compared with control schools [23]. The results demonstrated that the intervention approach is highly feasible, acceptable and holds significant promise for reducing the mean energy of foods packed in school lunch boxes.

Therefore, the primary aim of this study is to assess, via a Type I hybrid effectiveness-implementation cluster RCT [24], the effectiveness and cost-effectiveness of an intervention that makes use of an existing school mobile communication app (SkoolBag) to decrease the energy (kJ) content from discretionary foods and drinks packed in school lunchboxes. The intervention will also assess a range of implementation outcomes including school and parent level adoption, acceptability, feasibility and appropriateness in order to inform strategies for large scale dissemination.

## Methods

### Design

The cluster RCT will be conducted in 36 primary schools (for children aged 5–12 years). A hybrid Type I effectiveness-implementation design will assess intervention effects on dietary related outcomes and factors to inform future dissemination [24].

The research will be conducted and reported in accordance with the requirements of the Consolidated Standards of Reporting Trials (CONSORT) Statement [25]. Primary schools will be randomised to receive either the multi-component m-health lunchbox intervention, or to a control arm (standard school practices) after baseline data collection.

The primary outcome, mean energy (kJ) content of discretionary lunchbox foods and drinks packed in lunchboxes, will be observationally assessed at baseline and immediately post intervention (5 months or 1.5 school terms).

The secondary outcomes:

- **Food packed in the lunchbox:** The 1) mean total energy (kilojoules), 2) mean energy (kilojoules) from healthy foods and 3) mean percentage of lunchbox energy (kilojoules) from discretionary and healthy foods and drinks ***packed in the lunchbox*** will be observationally assessed for consenting students across Kindergarten to Year 6 in both groups at baseline and immediately post intervention (5 months).
- **Lunchbox food consumed at school:** The mean energy content of 4) discretionary food and drinks, 5) healthy food and drinks, 6) mean total energy (kilojoules) of foods and drinks and 7) mean percentage of lunchbox energy (kilojoules) from discretionary and healthy foods and drinks ***consumed at school*** will be observationally assessed for consenting students across Kindergarten to Year 6, from one randomly selected class from each year, in both groups at baseline and immediately post intervention (5 months).
- **Food consumed outside of school:** For exploratory purposes and to assess any compensatory behaviours, a sub sample of students’ intake outside of school hours and mean overall daily nutrient intake will also be assessed by parents via a short telephone survey, in addition to self-report [26] for consenting students from Years 5 and 6, at baseline and immediately post intervention at 5 months.
- **Implementation outcomes:** School and parent acceptability, feasibility, adoption and appropriateness will be assessed immediately post intervention (5 months). School engagement will also be measured and a cost-effectiveness evaluation will be conducted subject to assessment of efficacy.

The trial has been prospectively registered (ACTRN12618001731280).

### Setting

The study will be conducted across three local health districts (LHD) in New South Wales (NSW), Australia (Hunter New England (HNE), Central Coast (CC) and Mid North Coast (MNC)). These districts encompass major city, regional and remote areas and comprise 19% of the NSW population [27].

### Sample and participants

Thirty six primary schools located in three LHDs who are current users of the required school mobile communication app (SkoolBag) will be randomised to receive a 5 month (two school terms), multi-component lunchbox intervention or to a control arm (18 schools per arm). The intervention will be delivered to all students attending intervention schools from Kindergarten to Year 6.

### Recruitment

#### Schools

**Fig. 1:**
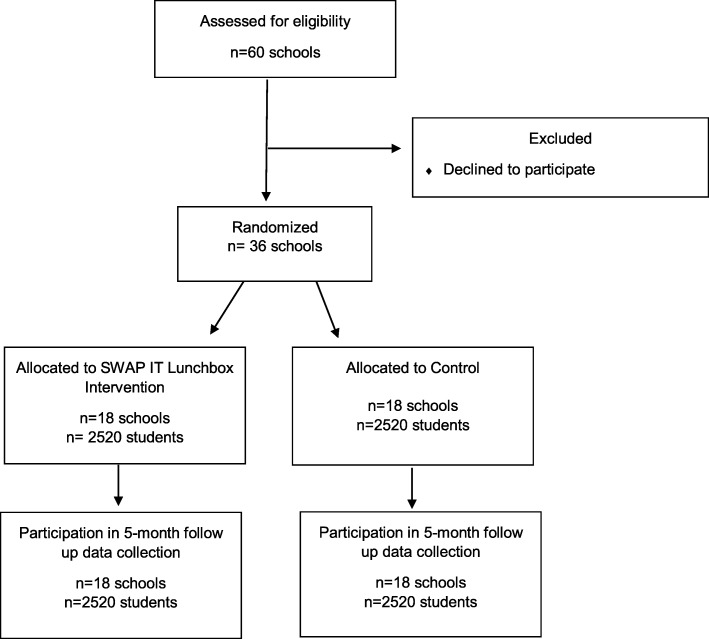
Consort flow diagram

Primary schools catering for students from Kindergarten to Year 6 will be eligible for inclusion if they meet the following criteria: Government schools; located in one of the participating LHD; greater than 120 student enrolments; current users of the preferred school mobile communication app (SkoolBag); and not participating in other nutrition based research studies. Central and secondary schools catering for students aged 13–18 years and schools primarily catering for children with special needs (such as intellectual disabilities) will be excluded. Eligible schools will be randomly ordered using a random number generator in Excel to randomise the order of schools approached and sent a letter of invitation. One week following the invitation, a member of the research team will contact the principal via telephone and seek consent. A face-to-face meeting will also be offered to all schools to outline the requirements of the study. Recruitment will continue until 36 schools consent to participate (see Fig. 1).

#### Parents and children

The child/parent recruitment strategy was developed based on our pilot study and reviews of evidence for facilitating participation in school-based research [23, 28]. Following recruitment of schools, all parents with a child enrolled in Kindergarten to Year 6 in consenting schools will be invited to participate in the study evaluation measures. Students will be provided with an information package containing an outline of the study and a consent form. Active parental consent will be required for child participation and parents will be asked, via the consent form, if they are an active user (i.e. downloaded the app) of the school communication app. One week following distribution of the information package, parents who have not returned a consent form will be telephoned by school-employed staff and asked if their child can participate in evaluation measures. A replacement consent form will be sent to parents who provide verbal consent. The research team have experience in recruiting schools and parents using such methods, obtaining response rates of 70–80% in previous trials [29].

### Randomisation and blinding

Following baseline data collection, schools will be randomly allocated to intervention or control via block randomisation on a 1:1 ratio using a computerised random number function. Randomisation will be stratified by the socioeconomic status of school locality using the Socio-Economic Index for Area (SEIFA 2006), as socioeconomic status is associated with child dietary intake [30, 31]. Randomisation will be undertaken by a statistician not involved in contacting schools in the study intervention or assessment. Data collectors and lunchbox analysis dietitians will be blinded, but school principals will be notified of their group allocation.

### Intervention

The intervention, primarily based on the pilot intervention was co-developed by a multidisciplinary team comprised of academic and end-user stakeholders from government health agencies, educational systems employees, universities and technology partners and parent representatives with expertise in nutrition, school based health intervention, behaviour change, implementation science and technology based interventions.

The intervention was developed using the Behaviour Change Wheel (BCW) [32]. The BCW consolidates 19 frameworks of behaviour change and provides a process to move from behavioural diagnosis to intervention design. The BCW recommends thorough formative evaluation to identify impediments to behaviour change and to select appropriate behaviour change techniques to address these. Accordingly, extensive formative research was conducted to identify barriers and potential behaviour change techniques that may support parents to pack healthy school lunchboxes. Specifically, the team undertook a review of published literature of existing lunchbox interventions [20], conducted focus groups with parents to identify local contextual barriers, and undertook telephone interviews with parents (*n* = 228) (Janssen, Sutherland, Nathan, Wyse, Lecathelinais, Finch, Wolfenden: Parent acceptability of using a mobile phone application to promote healthy lunchboxes for childcare- and school-aged children, unpublished) and principals (*n* = 196) [33] to assess barriers, and the acceptability of potential intervention strategies, content and delivery modalities. Based on such evidence, the BCW was used to select the most promising intervention strategies (behaviour change techniques) to address the most pertinent barriers to packing healthy lunchboxes.

Table 1 outlines the barriers identified via the formative research phase.

**Table 1:** Identified barriers to packing a healthy lunchbox

	Setting (school)	Family (parental)	Individual (child)
Barriers	• Food safety regarding lack of refrigeration and reheating facilities at school [34, 35] • School allergy policy [35] • Accessibility of fresh produce [35] • Availability of discretionary food and drink products [36] • Advertising and packaging [34, 36]	• Time to prepare lunchbox [34] • Cost and affordability and perception that healthy food is more expensive [34] • Poor nutritional knowledge on healthy food products [34] • Negative parental modelling [37] • Conflicting food purchases [37] • Parental food preferences [37] • Lack of skills in cooking and preparing food [37] • Convenience [34, 37] • Discretionary food provision as a reward for good behaviour [37] • Lack of social support [36] • Social norms and cultural challenges [36]	• Child preference and fussy eating [34] • Attitude [36] • Food and beverage likes and dislikes [36] • Parental pressure to offer what the child prefers [36]

**Fig. 2:**
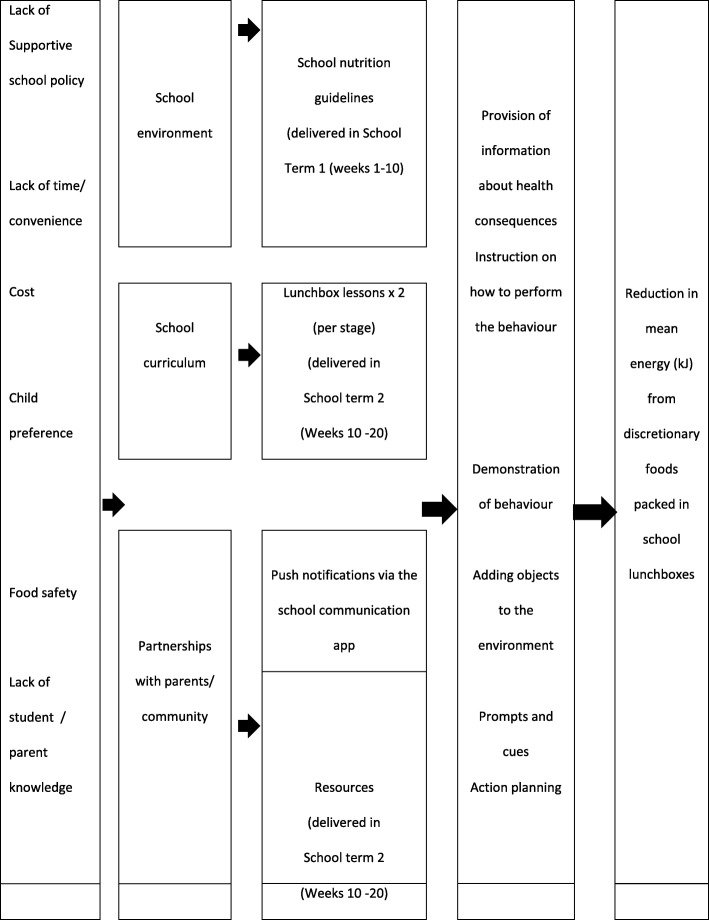
SWAP IT logic model

Minor modifications to the SWAP IT intervention were made following the pilot RCT which established potential efficacy, feasibility and acceptability in 12 Catholic primary schools, including minor wording and formatting changes to the push notifications, updates to the associated webpage, parent resource and flipcharts to include Aboriginal artwork and updated graphics and short professional learning module for school staff. A description of the intervention logic model is provided in Fig. 2.

The SWAP IT intervention encourages ‘swaps’ from discretionary food items to guideline-based healthier alternatives known as ‘everyday’ foods, with the m-health component (weekly pushed messages to parents) delivered via an existing school mobile communication app, Skoolbag. Schools purchase the SkoolBag app for a nominal fee annually and require parents to download an android or IOS version (free for parents) to receive direct school communication such as school reminders and notes regarding school events and student progress.

The intervention involves:

1. *Lunchbox nutrition guidelines*: Participating schools will be asked to adopt and endorse lunchbox nutrition guidelines, consistent with the WHO and the NSW Department of Education Nutrition in Schools policy [38]. A guideline template will be provided to support schools to communicate to the school community. Principal endorsement of guidelines will be promoted to the school community via the SkoolBag app and school newsletters, utilising the authority of the school Principal, to communicate school expectations and normalise healthy lunchboxes. In the pilot, 85% of schools adopted and endorsed such guidelines. With support from a trained dietitian, the development of lunchbox nutrition guidelines will occur in the first term (5 school weeks) of the intervention.
2. *Weekly support messages*: An existing school mobile communication app (SkoolBag) will be used to communicate healthy lunchbox messages to parents/carers. Through the SkoolBag app, content to support the packing of healthy lunchboxes will be sent to parents/carers at weekly intervals over the second school term (10 weeks) via push notifications (electronic messages). The messages are designed to act as prompts and cues to reinforce packing of ‘everyday’ foods children enjoy, provide tips and suggestions to assist parents to pack ‘everyday’ foods that are quick, convenient and low cost, and connect parents with tools and resources to improve their knowledge and skills to swap out discretionary foods and pack ‘everyday’ foods. The messages and embedded videos provide simple instructions on swapping, demonstrate the time needed to pack healthy lunchboxes and assist parents to plan healthy lunchbox swaps. Distribution of the messages via SkoolBag will be managed by the project team, not requiring school time or resources. In the pilot intervention, all ten messages were delivered to all parent users of the SkoolBag app in all schools resulting in the delivery of 11,500 messages to parents [23].
3. *Resources for parents*: Links embedded in the app messages will connect parents to additional resources housed on the program website. These resources provide parents with information regarding health consequences, simple instructions on making healthy lunchbox swaps, and assist in lunchbox planning to address child preference, cost, convenience and food safety. Additional physical resources including a SWAP IT ideas booklet (lunchbox ideas), clear drink bottle for water and an ice-brick to support food safety concerns will also be provided to parents.
4. *Curriculum resources for schools*: Schools will be provided with a short teacher professional learning session outlining the rationale for the study and providing the skills and resources required to deliver the classroom curriculum lessons. Teachers will be provided complimentary stage appropriate curriculum resources and classroom flipcharts, developed by dietitians in consultation with teachers to align with syllabus outcomes to reinforce program messages. The resources are designed to reinforce healthy food preferences and improve students’ nutrition knowledge.

### Control

Schools allocated to the control group will participate in data collection only. They will have access to the app but none of the lunchbox intervention content.

### Data collection procedures and measures

All schools will be offered 1 day of teacher relief funding (AUD$400) to reimburse the school for their time in assisting with data collection over the intervention period.

### Primary outcome

Mean energy (kJ) content of discretionary foods packed from the school lunchboxes will be assessed using a valid and reliable lunchbox observational audit, known as the School Food Checklist (SFC), [39, 40] an approach successfully applied in the pilot and deemed highly feasible in assessing foods packed in the lunchbox resulting in 98% of lunchbox photographs being included in the analysis and will be extended to also assess consumption by taking a second photo at the end of the final school break period. On a randomly selected school day, prior to recess, lunch and in-class vegetable and fruit breaks (pre meal assessment) [41, 42], students will be asked to display their lunchbox on their desk and remove all lids from storage containers. To prevent response bias, parents or students will not be aware of the day of data collection. Trained research assistants will take a photograph of each student’s lunchbox at the beginning of the school day prior to any contents being consumed (“foods packed”) and will also write down any ambiguous items to aid analysis. Students will also be asked if they intend to purchase from the canteen that day.

Students will be asked to place unconsumed or partially consumed items back into their lunchbox, after each meal break (in-class vegetable and fruit breaks, recess and lunch). A second photograph will be taken following the final meal break (post meal assessment). Measures of energy consumption will be calculated by subtracting the energy content of foods and drinks remaining in student’s lunchbox at the post-meal assessment from the energy content of foods and drinks in the lunchbox during pre-meal assessment (“foods consumed”).

The SFC is a previously validated tool shown to be accurate and reliable in measuring energy from food and drinks for the Australian context. The SFC [39, 40] enables assessment of the kJ content and serving size for each lunchbox item. The checklist includes 20 food and drinks categories including main food items such as bread, fast food and leftovers/mixed dishes and snack items such as noodles, packaged snacks, biscuits and crackers, chocolate and lollies, cheese, eggs, dried fruit and nuts, muesli and fruit bars, cakes and buns, muffins and scones, pastries, desserts, yoghurt, fruit, vegetables, milk, soft drink, water and fruit juice. Foods in each category were included based on the frequency of consumption at school for children aged five to 15 years, according to the National Nutrition Survey 1995 [39] and the average kJ per category identified. The SFC will be used to identify the total lunchbox contents and food items in the lunchbox including the kJ content, number of ‘everyday’ or discretionary lunchbox items and the mean cost of lunchbox items. Everyday items refer to food and drink items that are part of the core food groups as determined by the Australian Dietary Guidelines and Australian Guide to Healthy Eating [4]. Food items classified as discretionary choices are items considered to be energy dense with minimal nutritional value such as cakes, chocolate, lollies, crisps, muesli bars and fast food.

Minor modifications were made to the SFC to separately categorise ‘everyday’ and discretionary food choices and updated to reflect the mean cost of lunchbox items at the time the pilot study was conducted (2017). Categories that required adjustment included: biscuit and crackers, cakes and buns, muffins and scones, desserts and packaged snacks. All foods in these categories were individually divided and categorised as an ‘everyday’ or discretionary food by consensus among dietitians. The serve size and kJ per serve information was obtained from FoodWorks Professional Edition V7 (version 7; Xyris Software, Highgate Hill, QLD, Australia), or if unavailable from FoodWorks, via a snack food database created for pre-packaged items. The snack food database was created by dietitians based on a significant array of pre-packaged snacks available in Australian supermarkets and included detailed nutrition information for each food item.

Trained dietitians will observe each school lunchbox photo and classify each food and drink item according to its SFC category and the serving size. To further aid this process, decision rules developed will be used to ensure standardisation of assessments. Following the analysis of the pre-meal lunchbox photo, dietitians will analyse the post meal photo.

Students who make canteen purchases on the day of data collection will be excluded from analysis. Primary outcome data will be collected at baseline and immediately post intervention (5 months).

### Secondary outcomes

a) mean total energy (kJ) packed within the lunchbox; b) mean total energy (kJ) consumed from the lunchbox; c) mean energy (kJ) from discretionary foods and drinks consumed within the lunchbox; d) mean energy (kJ) from healthy foods packed and consumed from the lunchbox and e) percentage of lunchbox energy from discretionary and healthy foods and drinks, both packed and consumed, will be assessed using the above mentioned measures and procedures. Measures relating to the food packed will be based on the first photograph taken at the beginning of the school day, prior to the consumption of any food items. Measures relating to consumption will be based on the second photograph of the day being taken, after all meal breaks have occurred and all uneaten food is placed back into the lunchbox container.

#### Student school engagement

As school engagement including behavioural, emotional and cognitive engagement is considered crucial for achieving positive academic outcomes [43], at baseline and follow-up, students in years 5 and 6, as part of their student survey, will be asked to complete selected items from the validated School Engagement Measure (SEM) – MacArthur [43]. The SEM is a 19 item survey which will provide a measure of students’ behavioural (5 items), emotional (6 items) and cognitive engagement (8 items) at school.

#### Student consumption of discretionary foods outside of school hours

Whilst the purpose of the SWAP IT intervention is to decrease the mean energy content of discretionary foods packed within a school lunchbox, to identify any compensatory nutrition behaviour occurring out of school hours, parents will be asked to report via a short telephone survey, at baseline and follow-up, on their child’s nutritional intake outside of school hours and on weekends. Measures will be taken from the NSW Schools Physical Activity and Nutrition Survey (SPANS 2015) [6]. Additionally, an exploratory sub study will be conducted to assess mean daily nutrient consumption in consenting students from Years 5 and 6, who reliably self-report [26] at baseline and immediately post intervention at 5 months using the Australian Child and Adolescent Eating Survey (ACAES) [26] completed in class time via a pen and paper survey and facilitated by a trained research assistant. Pen and paper surveys will be provided for students to complete taking approximately 40 min. This food frequency questionnaire is a valid and reliable tool to identify usual dietary consumption, mean energy and nutrient intake over the past 3 months, and percentage energy from food groups including discretionary foods to aid in characterising any intervention effects [26]. The ACAES has been validated in students aged between nine and 16 years from the Hunter region in NSW and recommended for use in intervention trials [26]. The measurement of student consumption is for exploratory purposes, not powered to detect effects and intended for descriptive purposes, to describe any changes in the dietary behaviour of children outside of school hours.

#### Implementation outcomes

Consistent with the hybrid design, to identify if the intervention was implemented as intended and to inform future scale-up efforts the following implementation outcomes will be measured, based on the Proctor et al. [44] taxonomy of implementation outcomes. This includes:

- ▪ *Acceptability*: At follow-up, school principals will be requested to participate in a short interview where they will be asked the acceptability of the SWAP IT intervention in their school. Via a telephone interview, parents will also be asked about the acceptability of SWAP IT, based on Acceptability of Intervention Measure (AIM) [45], developed by Weiner et al., a four-item valid and reliable scale. The research team have achieved high school (77.8–96.4%) [23] and parent (74%) consent rates for telephone surveys.
- ▪ *Adoption*: Via the Principal interview, principals will be asked if the school adopted the lunchbox nutrition guidelines and asked for evidence of promotion to the school community. Parents will be asked if they had made any healthy swaps in the lunchbox based on the information received via the SWAP IT intervention via the parent telephone interview.
- ▪ *Appropriateness*: At follow-up, intervention principals (interview) and parents (telephone survey) will be asked questions related to intervention appropriateness via the Intervention Appropriateness Measure (IAM), a four-item valid and reliable scale [45].
- ▪ *Feasibility*: At follow-up, intervention principals (via interview) and parents (via telephone survey) will be asked to complete the Feasibility of Intervention Measure (FIM), a four-item valid and reliable scale [45].
- ▪ *Fidelity:* Project records, app analytics, as well as post-intervention measures completed by intervention principals (interview) and parents (telephone survey) will be used to determine the proportion of schools and parents that received and attended to each of the implementation strategies.
- ▪ *Implementation cost:* see cost and cost-effectiveness measure below.
- ▪ *Penetration:* Penetration will also be measured via reviewing app analytics, via the number of teachers who use the curriculum lessons within class and via the percentage of student consenting to participate in the evaluation component of the program.
- ▪ *Identification, measurement and valuation of resource use*: Resource use data pertaining to the development and implementation of the intervention will be collected prospectively using a bespoke cost capture tool developed in MS Excel (2013). Costs will be denominated in 2019/20 Australian dollars ($). Labour time and materials will be valued using market rates. Additionally, the cost of food and drink items packed within lunchboxes will be included in the SFC.
- ▪ *Cost-Effectiveness and budget impact analysis*: Subject to assessment of efficacy, a cost-effectiveness analysis will be conducted from multiple stakeholder perspectives comparing the intervention to a usual practice counterfactual. The primary cost-effectiveness outcome will be incremental cost per unit change in mean energy content of foods. In addition, the public finance budget impact to scale-up the SWAP IT intervention across NSW will be modelled.

### Sample size and power calculations

Based on our pilot results, a standard lunchbox contains 1089 kJ (SD = 900 kJ) from discretionary foods. With an ICC of 0.05, 36 schools with 140 students per school will enable detection of a 200 kJ difference between groups at follow-up on the primary trial outcome, with 80% power at the 0.05 significance level. As approximately 420 kJ across a whole day has potential to reduce the prevalence of childhood obesity [18, 19], and a child consumes a third of their daily energy requirements whilst at school [11], this magnitude of effect is considered meaningful at a population level.

### Analysis

All statistical analyses will be performed using SAS (version 9.3) statistical software. Using intention to treat principles, differences between groups in both costs and outcomes will be assessed using hierarchical linear (or logistic for binary outcomes) regression models, adjusting for pre-specified prognostic variables and random effects for repeated measures on students. Missing data will be imputed using multiple imputation methods. All statistical tests will be two tailed with an alpha of 0.05. Students intending to purchase their lunch from the canteen will be removed from the primary analysis to focus on students whose lunchbox is their sole source of energy for the day [23].

### Trial discontinuation or modification

It is not anticipated that any events would occur that would warrant discontinuing the trial. Any unforeseen adverse events will be reported to the Hunter New England Human Research Ethics Committee (primary approval committee) and advice sought regarding required action. The trial registration record will be updated with any protocol modifications and any deviations from original protocol will be reported in study outcome papers.

## Discussion

There is currently scarce evidence available regarding the effectiveness and cost effectiveness of interventions to support parents and primary schools in improving the nutritional quality of the foods packed in school lunchboxes. Internationally, there have been few RCTs with only five interventions published that have aimed to improve the nutritional quality of school lunchboxes and those conducted have largely failed to reach parents, were resource intensive and logistically challenging to deliver at scale [20]. The SWAP IT trial will be the first m-health based trial to assess the effectiveness of a lunchbox intervention targeting primary schools, children and their parents. A pilot of the intervention [23] indicated it to be highly feasible, acceptable to both parents and schools, and showed promising signs in reducing mean energy content of packed foods at a population level.

With scalability at the forefront of the intervention design, SWAP IT has been designed in collaboration with all key stakeholders and in partnership with key industry organisations in order to meet a policy objective to impact on childhood overweight and obesity at a population level. Using technology as the primary mode of delivery into the home environment, the intervention has been designed to overcome the barriers identified in the literature in improving the nutritional quality of lunchboxes and provides a means for reaching large number of parents. However, as the intervention is embedded within existing school communication systems and processes with which both schools and parents are actively engaged, findings of the pilot found that exposure to the intervention continues across the delivery period. Embedding behaviour change interventions within existing technological infrastructure provides a unique platform to scale-up the SWAP IT intervention, if shown to be effective.

## Data Availability

The datasets analysed during the study can be made available from the corresponding author on reasonable request, once the study is completed. All data will be stored securely as per ethical requirements. All participants will be issued a unique identification number following consent for confidentiality. The final trial dataset will be stored securely and accessed only by the study statistician. The results of this trial will be disseminated via publication in peer reviewed journal, conference presentations and reports to schools and relevant health and education departments.

## Abbreviations

ACAES: Australian Child and Adolescent Eating Survey
BCW: Behaviour Change Wheel
CC: Central Coast
HNE: Hunter New England
kJ: Kilojoules
LHD: Local Health District
MNC: Mid North Coast
NSW: New South Wales
RCT: Randomised Controlled Trial
SEIFA: Socio-Economic Index for Area
SFC: School Food Checklist
